# Semantic Size of Abstract Concepts: It Gets Emotional When You Can’t See It

**DOI:** 10.1371/journal.pone.0075000

**Published:** 2013-09-25

**Authors:** Bo Yao, Milica Vasiljevic, Mario Weick, Margaret E. Sereno, Patrick J. O’Donnell, Sara C. Sereno

**Affiliations:** 1 School of Psychology, University of Kent, Canterbury, United Kingdom; 2 School of Psychological Sciences, University of Manchester, Manchester, United Kingdom; 3 Behaviour and Health Research Unit, Institute of Public Health, University of Cambridge, Cambridge, United Kingdom; 4 Department of Psychology, University of Oregon, Eugene, Oregon, United States of America; 5 School of Psychology, University of Glasgow, Glasgow, United Kingdom; 6 Institute of Neuroscience and Psychology, University of Glasgow, Glasgow, United Kingdom; Utrecht University, Netherlands

## Abstract

Size is an important visuo-spatial characteristic of the physical world. In language processing, previous research has demonstrated a processing advantage for words denoting semantically “big” (e.g., *jungle*) versus “small” (e.g., *needle*) concrete objects. We investigated whether semantic size plays a role in the recognition of words expressing abstract concepts (e.g., *truth*). Semantically “big” and “small” concrete and abstract words were presented in a lexical decision task. Responses to “big” words, regardless of their concreteness, were faster than those to “small” words. Critically, we explored the relationship between semantic size and affective characteristics of words as well as their influence on lexical access. Although a word’s semantic size was correlated with its emotional arousal, the temporal locus of arousal effects may depend on the level of concreteness. That is, arousal seemed to have an earlier (lexical) effect on abstract words, but a later (post-lexical) effect on concrete words. Our findings provide novel insights into the semantic representations of size in abstract concepts and highlight that affective attributes of words may not always index lexical access.

## Introduction

Size is one of the most important properties of the physical world. Size affects the physics and biology of the world around us (e.g., [1], [2]). Size is one of the few dimensions that is iconically gestured during spontaneous speech (e.g., [3]). Recent advances in visual neuroscience have demonstrated category selectivity for object size along the ventral temporal cortex (e.g., [4]). While there is robust evidence that humans possess perceptual (e.g., visual) systems specialized for the processing of physical or “real-world” size, the involvement of these systems in language processing remains less well understood.

There is a growing body of evidence, however, suggesting that the semantic representation of physical size is automatically activated during visual word recognition. Rubinsten and Henik [5] demonstrated a size-congruency effect for animal name pairs that were visually presented in different font sizes (e.g., *ANT*-*lion* or *ant-LION*). Participants judged which of the two words was larger in either physical or semantic size. In both judgments, reaction times (RTs) were faster with size-congruent (*ant-LION*) versus size-incongruent (*ANT-lion*) stimuli. Their findings indicated that lexically-associated size information interacted with the perception of physical size. Sereno, O’Donnell, and Sereno [6], [7] further explored semantic size effects during lexical access. Using a lexical decision task, they observed that individuals were faster to recognize words representing big (e.g., *ocean*, *dinosaur*, *cathedral*) as opposed to small (e.g., *apple*, *parasite*, *cigarette*) items. Their findings suggested that size representations seem to be both automatically activated and differentially accessed.

Recent embodied or grounded cognition theories (e.g., [8]) provide a possible mechanism underlying the processing advantage for words with bigger semantic sizes. Such theories posit that there is an inextricable link between cognition and sensory-motor systems. According to these theories, language processing of words is thought to be grounded in mental simulations of semantically associated visuo-spatial representations. We would suggest that part of such representations must relate to real-world size, reflected by differential activations within the human visual system. For example, Murray, Boyaci, and Kersten [9] demonstrated that the degree of primary visual cortex activation depended on the perceived, not actual, size of a stimulus. Moreover, when viewed from the same distance, larger (as opposed to smaller) objects elicit more low spatial-frequency information which is transmitted faster through the magnocellular pathway (e.g., [10]). In word recognition, such information may become available faster via mental simulation for words representing larger objects, leading to a processing advantage over words representing smaller objects.

While representations of the semantic size of *concrete objects* can be embodied in visuo-spatial sensory processing, it is uncertain what can account for semantic size representations of *abstract concepts*. Unlike their concrete cousins, abstract concepts are not directly linked to our sensory-motor experiences of the physical world. Nonetheless, they can often be characterized in terms of size. Intuitively, we would classify concepts like *trust*, *eternal*, and *crisis* as “big”, and ones like *trace*, *impulse* and *humble* as “small”. A concept’s size can also vary depending on the context, as indicated in statements like, “*This is the biggest moment in my life*” or “*I like big ideas*”. The question remains, however, as to the representational nature of abstract size in language processing. The word *moment* does not refer to a physical entity and its size cannot be grounded in sensory-motor experiences in the same way as that of the word *horse* can. In this sense, the concept *moment* is neither big nor small.

Barsalou and Wiemer-Hastings [11] extended their account of knowledge representations to abstract concepts by suggesting that abstract meanings are captured in a repertoire of situational events and introspections. They proposed that while concrete concepts focus on objects in specific situations, abstract concepts rely on a broader range of components including introspective information such as emotions. This idea was recently supported and extended by Kousta, Vigliocco, Vinson, Andrews, and Del Campo [12]. They suggested that abstract concepts are more emotionally charged than concrete ones, which gives the former a residual processing advantage when imageability and contextual availability are controlled. They proposed that emotion plays an important role in acquiring, representing, and processing abstract concepts and that the lack of mappings from abstract words to the physical world is compensated for by internal mappings in the form of affective associations. Consequently, it is plausible to posit that the concept of size for abstract words may be represented through such affective associations. It is widely accepted that emotion can be characterized in a two-dimensional framework of arousal and valence (e.g., [13]–[16]). Arousal is a physiological and psychological state of alertness that varies in magnitude with the intensity of the experience. Valence indexes the inherent attractiveness or aversiveness of an entity and describes the polarity (positive or negative) of affective representations. More recently, event-related potential studies investigating how emotion words are processed as a function of their concreteness have demonstrated differential processing [17], [18]. The relationship between the dimensions of emotion and semantic size, however, has not to our knowledge been explored.

In the current study, we first extended previous research by examining the effects of semantic size on the recognition of concrete as well as abstract words. Second, we explored the relationship between semantic size and affective characteristics of words (arousal and valence) as well as the impact of these variables on lexical access. We hypothesized that responses would be faster to words denoting bigger objects/concepts (e.g., *elephant*, *paradise*) than to words denoting smaller objects/concepts (e.g., *ornament*, *intimate*) when variables such as word frequency, age of acquisition, and word length were controlled. This was supported by the observed processing advantage for bigger (concrete) words [6] as well as by a diverse literature which substantiates a “bigger is better” perspective (see, e.g., [19]–[22]). We also hypothesized that responses to concrete words would be faster than those to abstract words (see, e.g., [23]). Finally, we hypothesized that size representations of abstract concepts are more strongly tied to affective experiences than those of concrete concepts. That is, there should be a stronger link between semantic size and emotionality for abstract rather than concrete words. We first collected ratings on semantic size and affective characteristics for concrete and abstract words denoting big or small objects/concepts. Word recognition latencies were measured in a standard lexical decision task.

## Methods

All participants gave written informed consent and the experimental procedure was approved by the College of Science and Engineering Ethics Committee at the University of Glasgow.

### Participants

Sixty (34 female; age range 18–43 years, *M* = 22.75, *SD* = 4.25) members of the University of Glasgow community voluntarily participated in this study. All were native English speakers, had normal or corrected-to-normal vision, and had not been diagnosed with any reading disorder.

### Apparatus

The experiment was run on a Mac G4 (OS 9.0.4) computer, using PsyScope 1.2.5 PPC software [24]. Letter strings were presented on a Hansol 2100A 19″ color monitor (120 Hz, 1024×768 resolution) in 24-point Courier font (black letters on a white background). Participants sat at a viewing distance of around 32″ and approximately 3 letters subtended 1° of visual angle. Responses were made via a PsyScope Button Box and RTs were recorded with millisecond accuracy.

### Design and Materials

A 2 [Concreteness: Concrete vs. Abstract]×2 [Size: Big vs. Small] within-participant design was used. The experiment comprised a total of 220 words ranging from 4–8 characters in length. Half of the words had relatively concrete meanings (e.g., *castle*) while the other half had relatively abstract meanings (e.g., *wealth*). Within each Concreteness condition, half of the words described relatively big objects or concepts (e.g., *castle* and w*ealth*) while the other half described relatively small objects or concepts (e.g., *pocket* and *unique*).

Across all four conditions, words were matched on an item-by-item basis for word frequency (occurrences per million) and word length (number of letters). Word frequencies were obtained from the British National Corpus (BNC), a database of 90 million written word tokens (http://www.natcorp.ox.ac.uk). All word stimuli are listed in **Table S1**. Nonwords comprised 220 pronounceable, orthographically legal pseudowords (e.g., *zocker*) that were matched to word stimuli in terms of string length.

Ratings for all other psycholinguistic variables – concreteness, semantic size, emotional arousal, emotional valence, age of acquisition (AoA) – were collected from an independent sample in a computer-based rating task using a visual analogue scale (VAS). This was because such ratings for our stimulus set were not always available in existing databases or, in the case of semantic size, did not exist. We employed rating procedures similar to those used in the literature. Our specific procedures, instructions, and rating scales are detailed in **Procedure S1**. The specifications of the psycholinguistic variables for our materials across conditions are summarized in **Table 1**. Independent-samples *t*-tests run on the Concreteness and Semantic Size ratings showed that, subjectively, these manipulations were effective [Concreteness: *t*(218) = 41.61, *p*<.001; Semantic Size: *t*(218) = 28.02, *p*<.001]. That is, Concrete words (*M* = 88, *SD* = 7) were rated as being significantly more concrete than Abstract words (*M* = 35, *SD* = 12) and Big words (*M* = 70, *SD* = 9) were rated as being significantly bigger than Small words (*M* = 28, *SD* = 13).

**Table 1:** Specifications of the experimental words with standard deviations in parentheses.

	Concrete		Abstract	
	Big	Small	Big	Small
*N*	55	55	55	55
**Concreteness**	86.79 (8.36)	89.48 (4.44)	33.15 (10.86)	37.05 (11.91)
**Semantic Size**	67.58 (9.42)	22.05 (9.86)	72.34 (8.48)	33.99 (12.48)
**Arousal**	50.53 (15.07)	37.02 (11.07)	66.00 (9.53)	41.14 (14.27)
**Raw Valence**	54.41 (13.29)	54.12 (12.63)	55.02 (29.76)	46.25 (16.93)
**Absolute Valence**	33.77 (12.58)	28.72 (14.30)	62.55 (13.08)	37.15 (16.86)
**Age of Acquisition**	30.28 (10.68)	30.68 (9.89)	49.52 (16.40)	47.27 (15.47)
**Word Frequency**	29.10 (37.22)	29.83 (45.02)	27.25 (37.37)	26.94 (39.93)
**Word Length**	5.85 (1.25)	5.85 (1.25)	5.85 (1.25)	5.85 (1.25)

Ratings for the following factors were based on separate 100-point scales (low to high): Concreteness (abstract to concrete), Semantic Size (small to large), Arousal (unarousing to arousing), Raw Valence (negative to positive), and Age of Acquisition (early to late). Absolute Valence was calculated via the following transformations: (a) shifting the 0 to 100 scale to a −50 to +50 scale (to more appropriately represent valence); (b) taking the absolute value of each rating (resulting in a 50-point scale); and (c) doubling each value to obtain a 100-point scale (from low to high unsigned valence). Word Frequency is expressed in occurrences per million and Word Length in number of letters.

### Procedure

Participants were tested individually and the entire experiment lasted around a half hour. They were given a consent form and written instructions. They were told that half of the stimuli were words and half were nonwords and that their task was to press the corresponding response button as quickly and as accurately as possible. Participants were first presented with a practice block of 8 trials to become accustomed to the procedure. Each trial began with a blank screen for 1000 ms, followed by a centrally presented fixation cross for 200 ms. The cross was then replaced by another blank screen for 500 ms after which the letter string was presented centrally until the participant responded. Word responses were made using the right forefinger on the right (green) key of the Button Box, labelled “W,” and nonword responses with the left forefinger on the left (red) key, labelled “NW.” The experimental trials (220 words and 220 pseudowords) were presented in a different random order to each participant with three programmed breaks.

## Results

Three different types of analyses were performed on the data. In order to directly compare our results with those of Sereno et al. [6], we first assessed the effects of Concreteness and Size via within-participant analyses of variance (ANOVAs). We then performed correlational and multiple regression analyses to better understand the relationship between our factors of Concreteness and Size and the emotional dimensions (arousal and valence) of the stimuli. Finally, we employed moderated mediation analysis to aid in determining the dynamic interrelationship among these variables during word recognition.

### Extending the Size Effect from Concrete to Abstract Words

The mean RT and percent error (%Error) data (with standard deviations) are presented in **Table 2**. Our initial analysis adopted the same methods employed by Sereno et al. [6] so that direct comparisons could be made. After removing error trials (3.8% over all trials), the RT data were subjected to two trimming procedures (with an additional data loss of 1.9%). Items with RTs longer than 1500 ms or shorter than 250 ms were first excluded. For each participant in each condition, items having RTs beyond two standard deviations were additionally excluded. These procedures (error and outlier removal) resulted in an average RT data loss of 5.7% per participant.

**Table 2:** Mean RTs (in ms) and %Error (with standard deviations in parentheses) across experimental conditions.

	Big		Small	
	Concrete	Abstract	Concrete	Abstract
**RT**	542 (63)	564 (70)	556 (77)	582 (78)
**%Error**	2.3 (2.2)	4.1 (3.2)	2.8 (2.8)	5.9 (5.1)

For RT and %Error data, 2 [Concreteness: Concrete vs. Abstract]×2 [Size: Big vs. Small] ANOVAs were performed both by participants (*F*_1_) and by items (*F*_2_). For RT, the main effects of Concreteness and Size were both significant [Concreteness: *F*_1_(1,59) = 90.92, *p*<0.001, Cohen’s *f* = 1.24; *F*_2_(1,54) = 47.91, *p*<0.001, Cohen’s *f* = .89; min*F*′(1,100) = 31.37, *p*<.001; Size: *F*_1_(1,59) = 33.16, *p*<0.001, Cohen’s *f* = .75; *F*_2_(1,54) = 20.40, *p*<0.001, Cohen’s *f* = .61; min*F*′(1,105) = 12.63, *p*<.001]. As expected, responses to Concrete words (549 ms) were faster than those to Abstract words (573 ms); responses to Big words (553 ms) were faster than those to Small words (569 ms). The Concreteness×Size interaction was not significant [*F*s<1]. Thus, the processing advantage for Big over Small words was equally pronounced for Concrete and Abstract words. For %Error, as with the RT data, both main effects were significant [Concreteness: *F*_1_(1,59) = 52.24, *p*<0.001, Cohen’s *f* = .94; *F*_2_(1,54) = 10.90, *p*<0.01, Cohen’s *f* = .45; min*F*′(1,76) = 9.02, *p*<.01; Size: *F*_1_(1,59) = 9.50, *p*<0.01, Cohen’s *f* = .40; *F*_2_(1,54) = 7.09, *p*<0.05, Cohen’s *f* = .36; min*F*′(1,109) = 4.06, *p*<.05]. Participants made fewer errors in response to Concrete (2.6%) and Big (3.2%) words in contrast to Abstract (5.0%) and Small (4.4%) words, respectively. Although the interaction was significant by participants [*F*_1_(1,59) = 5.25, *p*<0.05], it was not by items [*F*_2_(1,54) = 1.18, *p*>0.25; min*F*′(1,77) = .96, *p*>.30].

Overall, our results consistently showed (orthogonal) processing advantages for Concrete over Abstract and for semantically Big over Small words. These advantages were reflected in faster recognition times and higher accuracy rates. The main effect of Concreteness is in line with past literature demonstrating that concrete words are generally processed faster than abstract words (e.g., [23], [25]–[27]). Likewise, the main effect of Size replicated previous findings by Sereno et al. [6]. While their stimuli were limited to concrete words, we found the same pattern of effects with abstract words.

The questions remain, however, as to why bigness confers a processing advantage to abstract concepts and what this might entail in terms of the nature of their underlying representations. As mentioned earlier, abstract concepts cannot be embodied in the same way as concrete objects in visuo-spatial modalities. To resolve this paradox, we conducted a series of correlation and regression analyses investigating the relationships between semantic size and emotion and how they might influence lexical access.

### Establishing the Relationships between Size, Concreteness, and Emotion

#### Size, arousal, valence, and concreteness

In our word specifications (Table 1), Big words tended to have higher emotionality (Arousal and Absolute Valence) than Small words. We explored the relationships between these variables by initially regressing Size on Arousal, Absolute Valence, and the Arousal × Absolute Valence interaction. The results are summarized in Table 3. We found that Arousal was the only significant predictor of Size (we obtained similar results when using Raw Valence values). We thus focused on Arousal as the dimension that may carry information about the size of concepts.

**Table 3:** Linear regression on semantic Size with Arousal, Absolute Valence, and their interaction term as predictors.

	B	95% CI		*p*	*r*	VIF
**Arousal**	**17.228**	[14.089	20.367]	**<0.001**	**0.732**	**2.096**
**Absolute Valence**	**0.565**	[−2.562	3.692]	**0.722**	**0.534**	**2.080**
**Arousal × Absolute** **Valence**	−**0.925**	[−3.185	1.335]	**0.421**	**0.161**	**1.086**

Reported are the slopes (Bs) for each regressor, their associated 95% confidence intervals (CIs), and *p*-values. Also shown are their zero-order correlation coefficients (*r*s) and variance inflation factors (VIFs; a VIF indexes the extent to which the variance of an estimated regression coefficient is increased because of collinearity).

Next we examined whether the correlation between Size and Arousal varied as a function of Concreteness. We hypothesized that representations of size for abstract words may be more strongly grounded in introspections and emotions. Such grounding may be weaker in concrete words as an object’s size is presumably linked more directly to visuo-spatial representations. We conducted a regression on Size with Concreteness, Arousal, and their interaction as predictors. The results are summarized in **Table 4**. Overall, it showed that the correlation between Size and Arousal was not significantly moderated by Concreteness.

**Table 4:** Linear regression on semantic Size with Concreteness, Arousal, and their interaction as predictors.

	B	95% CI		*p*	*r*	VIF
**Concreteness**	.746	[−1.608	3.100]	**0.533**	−**0.256**	1.177
**Arousal**	17.682	[15.287	20.077]	**<0.001**	**0.732**	1.218
**Concreteness ×** **Arousal**	.041	[−2.170	2.252]	**0.971**	−**0.138**	1.038

Reported are the slopes (Bs) for each regressor, their associated 95% confidence intervals (CIs), and *p*-values. Also shown are their zero-order correlation coefficients (*r*s) and variance inflation factors (VIFs).

The Size-Arousal correlation supported our hypothesis that the semantic size of abstract concepts may be represented via emotional content. It was, however, unexpected that the size associated with concrete words was correlated with emotional arousal to a similar extent, as we had originally assumed that the size of concrete objects is grounded in visuo-spatial representations. Theoretically, such a Size-Arousal correlation for concrete entities could imply two types of relationships. One possibility is that Size and Arousal share a representational nature and that the concept of size may be represented in the form of emotional arousal, in the same way we have stipulated for abstract concepts. The other is that Size and Arousal are two independent constructs that are linked. That is, the activation of size representations (e.g., visuo-spatial representations) during lexical access of concrete objects elicits a subsequent emotional response of arousal. This would also result in a significant correlation between the two.

We reasoned that one way to distinguish between these two underlying relationships is to examine the effects of Size and Arousal on word recognition latencies. The first account (H1) assumes that Arousal underlies the semantic representations of Size and, hence, should be activated *during* lexical access. It predicts that Arousal should index the relative speed of word recognition *interchangeably* with Size. The second account (H2) posits that emotional arousal is elicited subsequently *after* lexical access. It predicts that Arousal should affect word recognition latencies *independently* from Size. To test these accounts, we carried out multiple regression analyses and examined the effects of Size and Arousal on RTs.

#### Effects of size and arousal on word recognition latencies

Data preparation involved first removing trials with incorrect responses (3.76% of the data) and then those with RTs longer than 1500 ms or shorter than 250 ms (a further 0.99% of the data). In total, 12573 trials (95.25% of the data) were submitted to the multiple regression analyses.

We conducted the multiple regressions in two rounds to account for between-participant variability. A first round of analyses was performed to assess individual participants’ sensitivity to the lexical variables included in the model. We used a full regression model investigating all possible main effects and interactions between Concreteness, Size, and Arousal. The regression results are presented in **Table 5**. We standardized the variables to minimize multicollinearity and computed the corresponding variance inflation factors (VIFs) as collinearity diagnostics. Regression weights (Bs) index the strength of each regressor (main effects or interaction term) on participants’ response times. Steeper slopes imply that RTs are modulated to a greater extent by these lexical variables individually and/or interactively. We also calculated semi-partial correlation coefficients to estimate the effect size of each regressor. As the slopes and the semi-partial correlation coefficients for each regressor had been calculated for each participant, a second round of analyses was then carried out to assess whether these slopes (i.e., correlation strengths) and semi-partial correlation coefficients (i.e., correlation relevance) were consistently different from zero across all 60 subjects. We performed a percentile bootstrap with alpha set to 0.05 using 5000 samples with replacement to calculate the 95% confidence intervals (CIs) and associated *p*-values [28].

**Table 5:** Multiple regression results.

Predictor	B	95% CI		*p*	FDR	R^2^(%)	95% CI (%)		VIF	
**Concreteness**	−11.534	[−15.019	−8.157]	0	1	0.75	[0.50	1.07]	2.081	
**Size**	−11.684	[−16.707	−6.859]	0	1	0.72	[0.52	0.93]	3.052	
**Arousal**	−4.347	[−8.738	0.031]	0.052		0.48	[0.31	0.67]	2.789	
**Concreteness × Size**	0.131	[−3.776	4.250]	0.948		0.45	[0.30	0.61]	2.737	
**Concreteness × Arousal**	2.365	[−1.488	6.227]	0.234		0.45	[0.31	0.60]	2.261	
**Size × Arousal**	2.926	[−0.172	5.974]	0.063		0.49	[0.33	0.67]	1.286	
**Concreteness × Size × Arousal**	−6.780	[−10.161	−3.451]	0	1	0.44	[0.31	0.58]	2.243	
**Intercept**	582.736									

Reported are the slopes (Bs) for each regressor, the associated 95% confidence intervals (CIs), *p*-values, and whether they survived the False Discovery Rate (FDR) correction (*p*<0.05) for multiple comparison (significant effects are marked with 1s). Also reported are the regressors’ semi-partial correlation coefficients (R^2^s), the associated 95% CIs, and variance inflation factors (VIFs).

**Figure 1:**
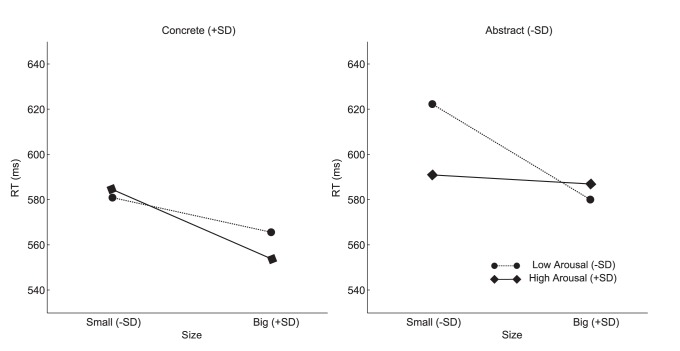
The Concreteness × Size × Arousal interaction. The left panel illustrates the Size × Arousal interaction at a high concreteness rating level (M+SD). The right panel illustrates the same interaction but at a low concreteness level (M−SD). The dotted lines with circles at both ends represent a low arousal level (M−SD). The solid lines with diamonds at both ends represent high arousal level (M+SD). The slopes of the two lines indicate the strength and direction of the Size effects on RTs at the different levels of Arousal.

In line with our ANOVA results, we observed significant main effects of Concreteness and Size. Both main effects displayed negative effects on RTs – that is, RTs were faster with higher values of either Concreteness or Size (i.e., more concrete or semantically bigger words). There was also a significant Concreteness × Size × Arousal interaction. We initially explored this interaction by observing the Size × Arousal interaction at putative high and low concreteness levels (i.e., “concrete” and “abstract” words, with concreteness ratings of M+SD and M-SD, respectively). The Size effects were reflected in the slopes (Bs) at putative “high” (M+SD) and “low” (M−SD) arousal levels. The results are summarized in **Table 6** and illustrated in **Figure 1**. Size effects were consistently robust in all conditions except for “abstract” words of “high” arousal.

**Table 6:** Summary of the Size effects (slopes) at putative high and low levels of Concreteness and Arousal.

Predictor		B (Size)	95% CI		*p*	Intercept
**“Concrete”**	**“Low” Arousal (M**−**SD)**	−7.699	[−12.754	−2.745]	0.002	573.184
**(M+SD)**	**“High” Arousal (M+SD)**	−15.406	[−23.115	−8.134]	<0.001	569.220
**“Abstract”**	**“Low” Arousal (M**−**SD)**	−21.521	[−31.056	−12.118]	<0.001	600.982
**(M**−**SD)**	**“High” Arousal (M+SD)**	−2.109	[−10.580	6.079]	0.628	587.559

Reported are the slopes (Bs) for each regressor, the associated 95% confidence intervals (CIs), *p*-values, and intercepts.

To statistically assess the significance of the Size × Arousal interaction in concrete and abstract words, a median split of the RT data based on Concreteness was taken, and the same regression analysis was performed on RTs with Size, Arousal, and the Size × Arousal interaction as predictors of RTs. These results are summarized in **Table 7**. At lower levels of concreteness, there was a significant Size × Arousal interaction. The main effect of Size, however, did not survive the FDR correction. At higher levels of concreteness, only the main effect of Size was significant.

**Table 7:** Multiple regression results using a median split of Concreteness.

	Predictor	B	95% CI		*p*	FDR	R^2^(%)	95% CI (%)		VIF
	**Size**	−11.240	−16.400	−6.341	0.000	1	1.21	0.80	1.66	2.269
**Concrete**	**Arousal**	−1.065	−5.117	3.043	0.576		0.72	0.50	0.99	2.328
**words**	**Size × Arousal**	−1.047	−4.917	2.877	0.604		0.91	0.60	1.27	1.083
	**Intercept**	570.114								
	**Size**	−7.810	−15.342	−0.216	0.044		1.09	0.77	1.45	2.269
**Abstract**	**Arousal**	−7.240	−15.138	0.606	0.064		1.17	0.83	1.54	2.328
**words**	**Size × Arousal**	5.043	1.433	8.783	0.004	1	0.84	0.60	1.14	1.083
	**Intercept**	595.721								

Reported are the slopes (Bs) for each regressor, the associated 95% confidence intervals (CIs), *p*-values, and whether they survived the False Discovery Rate (FDR) correction (*p*<0.05) for multiple comparison (significant effects are marked with 1s). Also reported are the regressors’ semi-partial correlation coefficients (R^2^s), the associated 95% CIs, and variance inflation factors (VIFs).

Overall, the results showed that, for abstract words, Size and Arousal influenced word recognition latencies interactively. Specifically, the Size effect was salient at lower levels of arousal, but was masked at higher levels (**Figure 1**, right panel). Critically, Size and Arousal appeared to act competitively. Such a result pattern favors our first hypothesis (see H1) to account for the correlation between Size and Arousal which suggests that Size and Arousal share a common representational nature. In contrast, for concrete words, Size alone influenced lexical access, although the Size effect was numerically enhanced with higher levels of arousal (**Figure 1**, left panel). This pattern was in line with our second hypothesis (see H2) which suggests that Arousal is an independent construct that can be subsequently elicited by the activation of visuo-spatial (Size) representations and, hence, does not directly drive lexical access. To further validate these speculations, we carried out a series of moderated mediation analyses.

### Evaluating the Contributions of Arousal to the Size Effect in Concrete and Abstract Words

Mediation, or an indirect effect, is a mechanism or process underlying an observable relationship between a dependent variable *Y* and an independent variable *X* where the effects of *X* are transmitted by a *mediator M* onto *Y*. In other words, *X* predicts *Y* because *X* affects *M* and *M* affects *Y*. Moderated mediation (i.e., a conditional indirect effect) refers to a mediation effect that is dependent on different levels of a *moderator W*. If the moderator *W* were gender (with levels male and female), an example of moderated mediation would be that *M* mediates *X*→*Y* in males but not in females (for an explanation of moderated mediation, see [29], [30]).

**Figure 2:**
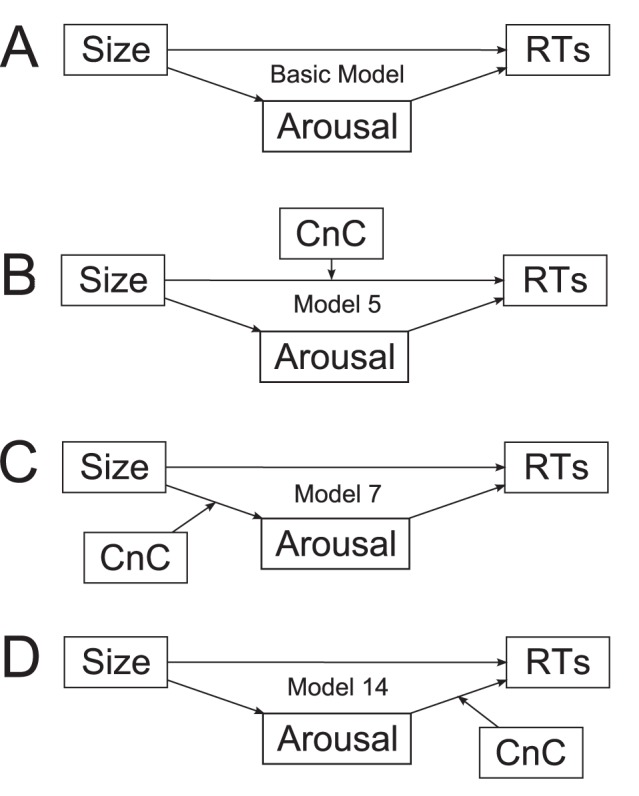
Schematic illustrations of the moderated mediation models [29], [30] under testing. Panel A illustrates the basic mediation model where Size can either directly or indirectly influence RTs via Arousal. Panel B, C, and D illustrate three possibilities where Concreteness (CnC) can moderate the direct or indirect effect of Size on RTs. The relative spatial layout does not imply an absolute time frame for processing. Our analyses lend greatest support to Model 14 (Panel D).

The current moderated mediation analyses employed the bootstrapping technique of Hayes ([31]; PROCESS macro Beta release 130612, Models 5, 7, and 14). The three models under testing, presented in **Figure 2**, were based on a simple mediation model (**Figure 2A**; Model 4 in PROCESS) in which Size has a direct effect on RTs and an indirect effect on RTs via Arousal. We probed the moderation (i.e., conditional) effect of Concreteness (CnC) on the direct pathway from Size→RTs (Model 5; **Figure 2B**), as well as on the indirect pathways, from Size→Arousal (Model 7; **Figure 2C**) and from Arousal→RTs (Model 14; **Figure 2D**). Recall, we hypothesized that Size effects on RTs may be mediated via Arousal in Abstract but not in Concrete words. Thus, we predicted that the Concreteness moderation effects should mostly likely be observed on the path Arousal→RTs (Model 14) and possibly on the path Size→RTs (Model 5). It would unlikely be observed on path Size→Arousal (Model 7) as it was already demonstrated that Size was consistently predicted by Arousal independent of Concreteness (see **Table 4**).

The data preparation was identical to that used in our multiple regression analyses and valid trials were submitted to PROCESS. The PROCESS macro was run on IBM SPSS Statistics 20. We employed 10,000 bootstrap re-samples with bias-corrected and bias-accelerated 95% confidence intervals (CIs) as recommended. Variables were centered before constructing the interaction terms to minimize multicollinearity.

**Figure 3:**
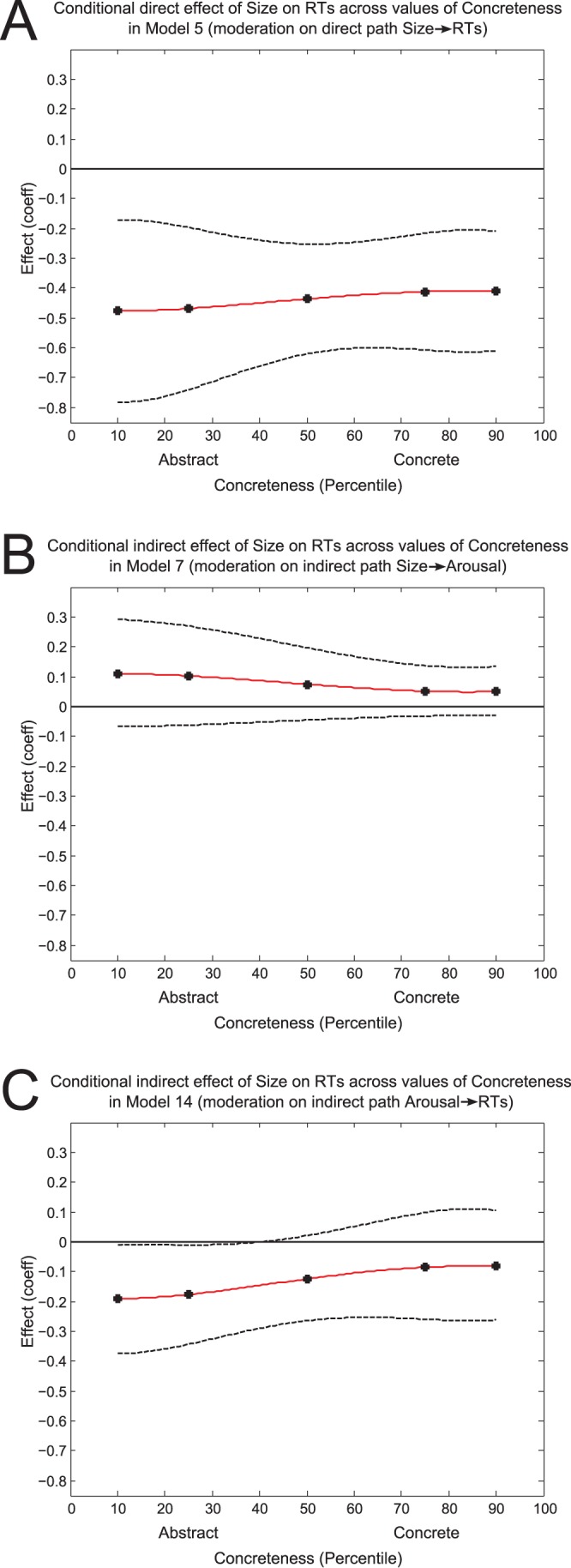
Illustrations of the moderation (conditional) effect of Concreteness by model. The solid red line represents the mean effect of Size across values of Concreteness. The five filled circles correspond to the mean Size effect at the 10^th^, 25^th^, 50^th^, 75^th^, and 90^th^ percentiles of the Concreteness ratings (see also Table 8). The upper and lower dotted lines represent the 95% confidence intervals around the means. The curves were fit using 3^rd^ and 4^th^ degree polynomial functions. A horizontal line crossing the 0 value on the y-axis is displayed as a reference point to visualize the significance of the effect. Panels A, B, and C correspond to Models 5, 7, and 14 (and Panels B, C, and D of Figure 2), respectively. The data pattern lends greatest support to Model 14 (Panel C).

The results are summarized in **Table 8** and the conditional effects are displayed in **Figure 3**. Overall, the results showed that the direct effect of Size→RTs was significant in all three models (*t*s<−3.07, *p*s<0.003). In Model 5 (**Figure 2B**, **Figure 3A**), this direct effect was significant at all levels of Concreteness (all CIs did not include 0), suggesting that it was not moderated by the latter. The indirect effects of Size→Arousal→RTs were significant when the CIs did not contain 0 [30]. Specifically, for Model 7 (**Figure 2C**, **Figure 3B**), this indirect effect was not significant at any level of Concreteness (all CIs included 0). Hence, the posited moderation of the Size→Arousal segment by Concreteness was not supported. In contrast, for Model 14 (**Figure 2D**, **Figure 3C**), Concreteness moderated the indirect effect of Size→Arousal→RTs. The indirect effect was significant in Abstract words (i.e., at the 10^th^ and 25^th^ percentile of the Concreteness distribution, the CIs did not contain 0), but not in Concrete words (i.e., at the 50^th^, 75^th^ and 90^th^ percentile of the Concreteness distribution, the CIs did include 0). In moderated mediation analyses, this kind of *conditional* indirect effect indicates the existence of a moderation effect [30].

**Table 8:** Results for moderated mediation analyses by model.

Model 5		Effect	SE	CI low	CI high	*t*	*p*
	**10^th^ Percentile**	−0.477	0.155	−0.781	−0.172	−3.071	0.002
**Direct effect**	**25^th^ Percentile**	−0.468	0.139	−0.740	−0.197	−3.381	0.001
**(Size**→**RTs)**	**50^th^ Percentile**	−0.436	0.094	−0.620	−0.252	−4.640	0.000
	**75^th^ Percentile**	−0.412	0.100	−0.607	−0.216	−4.132	0.000
	**90^th^ Percentile**	−0.409	0.102	−0.609	−0.210	−4.016	0.000
**Indirect effect**		−0.129	0.074	−0.274	0.015		
**Model 7**		**Effect**	**SE**	**CI low**	**CI high**	***t***	***p***
**Direct effect**		−0.469	0.092	−0.648	−0.289	−5.111	0.000
	**10^th^ Percentile**	0.110	0.092	−0.067	0.291		
**Indirect effect**	**25^th^ Percentile**	0.103	0.086	−0.062	0.271		
**(Size**→**Arousal**→**RTs)**	**50^th^ Percentile**	0.074	0.062	−0.045	0.195		
	**75^th^ Percentile**	0.052	0.044	−0.032	0.139		
	**90^th^ Percentile**	0.050	0.042	−0.031	0.134		
**Model 14**		**Effect**	**SE**	**CI low**	**CI high**	***t***	***p***
**Direct effect**		−0.427	0.091	−0.607	−0.248	−4.676	0.000
	**10^th^ Percentile**	−0.190	0.094	−0.374	−0.005		
**Indirect effect**	**25^th^ Percentile**	−0.176	0.084	−0.343	−0.010		
**(Size**→**Arousal**→**RTs)**	**50^th^ Percentile**	−0.124	0.072	−0.264	0.021		
	**75^th^ Percentile**	−0.085	0.091	−0.259	0.098		
	**90^th^ Percentile**	−0.082	0.093	−0.261	0.105		

Reported are the Effects (beta values), the bootstrap-estimated Standard Errors (SEs), and the lower and higher boundaries of the bootstrap-estimated Confidence Intervals (CIs). *t-* and *p-*values are also reported for direct effects.

The moderated mediation analyses indicated that semantic size of words *directly* influences lexical access speed in both Concrete and Abstract words. In the latter, this Size effect was also partially mediated through Arousal, thereby affecting lexical access *indirectly*. These results complemented our regression data and suggested again that in Abstract words, semantic size may be partially represented in emotional arousal, whereas in Concrete words, size may elicit activation of emotional arousal post-lexically (see **Figure 2D** and **Figure 3C**).

## Discussion

The current study examined whether semantic size of concrete as well as abstract words influenced their recognition speed in a lexical decision task. Results showed that words denoting bigger objects or concepts were recognized significantly faster than those indicating a smaller semantic size, irrespective of the concreteness of the entities. Regression analyses additionally revealed that semantic size was highly correlated with subjective ratings of emotional arousal. Our moderated mediation analysis, however, demonstrated that the effects of arousal contributed more centrally to the recognition of abstract in comparison to concrete words.

Overall, the present results replicated the previous findings by Sereno et al. [6] using a larger stimulus set (220 vs. 90 words) and extended the scope of semantic size from concrete objects to abstract concepts. The present results are compatible with the embodied cognition framework (e.g., [8]) in which cognition is grounded in bodily states, sensory-motor simulations, and situated action. Much research has demonstrated that language comprehension of concrete meanings leads to activation of associated sensory-motor cortices at both a lexical level (e.g., [32]–[34]) and a sentence/discourse level (e.g., [35]–[38]). Processing of concrete words should, by these mechanisms, lead to activation of associated visuo-spatial representations. Such representations may be accessed relatively faster in words denoting bigger objects [10], thereby resulting in a processing advantage over words denoting smaller objects.

With respect to abstract words, Barsalou and Wiemer-Hastings [11] proposed that abstract concepts and meanings are grounded in introspective states. They explored this idea by asking participants to generate features for highly concrete words (e.g., *bird*, *car*, *sofa*), highly abstract words (e.g., *truth*, *freedom*, *invention*), and intermediate words (e.g., *cooking*, *farming*, *carpeting*). They found that features for abstract concepts focused more on introspective and social content than on physical settings. Kousta et al. [12] further proposed that the lack of mappings from abstract words to the physical world may be complemented by mappings to the internal world in the form of affective associations. The present study can provide a substantive example of affective grounding for abstract words. Specifically, we showed that the semantic size of abstract concepts was partially grounded in emotional arousal and was automatically accessed during word recognition.

The question remains, however, as to why big abstract concepts are recognized faster. It is evident that emotion words are generally processed faster (e.g., [12], [39], [40]). Activation of higher arousal during word recognition may trigger a higher level of alertness and attention, resulting in faster response times. Nevertheless, this cannot fully account for the size effect on recognition latencies in abstract words. The direct effect of size remained significant regardless of its mediated pathway via arousal. Thus, while abstract size is *partially* represented in arousal, it may also be coded in other forms of representations, for example, the situational events and introspections that are associated with abstract meanings as suggested by Barsalou and Wiemer-Hastings [11]. Bigger concepts (e.g., *disaster*) tend to comprise a “bigger” range of introspective, social, and situational associations than smaller concepts (e.g., *incident*). Access to a richer network of semantic information grants bigger concepts a cognitive advantage over smaller concepts in word recognition (see also [23], for the context availability model and a similar contrast between concrete and abstract words). It is possible then to account for a significant direct effect of size in terms of such variations in the scale and density of semantic networks. Future research could test these speculations by examining the distribution of neural activity across the cortex during the processing of big versus small abstract words.

In a broader context, the current study also highlights the distinction between *intrinsically* and *extrinsically* emotional words. The former expresses or implies an emotional state (e.g., *panic*) while the latter elicits one (e.g., *shark*). Although affective characteristics can be similarly attributed to both categories of words, their role during lexical access may differ. That is, affective features are, by definition, more an integrated part of the semantic representations of intrinsically emotional words and more a semantic consequence of accessing extrinsically emotional words. Emotional attributes of words, hence, do not always index lexical access. This may account for the mixed results on affective word processing. In the emotion word literature, some studies demonstrate a processing advantage for positive over neutral words (e.g., [39]–[42]), some show an advantage for negative over neutral words (e.g., [17], [41], [43]), and others observe an advantage for positive over negative words (e.g., [44]–[46]). Such variability could potentially be due to differences in the ratio of intrinsically and extrinsically emotional words presented. Future research on affective word processing may consider explicitly distinguishing between the two types of words.

## Conclusions

Our results suggest that semantic size is automatically accessed when visually reading a word. Words having larger semantic sizes are activated more quickly for both concrete and abstract words. Although semantic size is highly correlated with emotional arousal, its effect was mediated via arousal in abstract but not in concrete words. This suggests that emotional arousal is an integrated part of semantic size in abstract words but may be elicited post-lexically by semantic size in concrete words. Further investigations of the mental representations of semantic size can use alternative measures such as eye movements during reading to rule out task effects or event-related brain potentials or BOLD signals during single word presentation to explore its underlying neural bases.

## Supporting Information

Table S1 **Word stimuli across conditions.**
(DOCX)

Table S2 **The five rating scales.** For each scale, the definition of each variable given in the instructions and the associated labels (from left to right) of each rating scale are indicated.
(DOCX)

Procedure S1 **Word rating task.**
(DOCX)
