# Supplementary material for: Semantic Size of Abstract Concepts: It Gets Emotional When You Can’t See It
Source: PLoS One. 2013 Sep 25;8(9):e75000. doi: 10.1371/journal.pone.0075000 (PMC3783453; doi:10.1371/journal.pone.0075000)
Supplement: Table S1 — Word stimuli across conditions. (DOCX) [file pone.0075000.s001.docx]

**Table S1. Word stimuli across conditions**

| **Concrete** | | **Abstract** | |
| --- | --- | --- | --- |
| **Big** | **Small** | **Big** | **Small** |
| stag | plum | zeal | meek |
| sofa | pill | roar | hush |
| lion | coin | bang | tidy |
| lake | seed | loud | hint |
| ship | bird | rich | pity |
| bear | spot | huge | rare |
| tree | page | wide | soon |
| farm | book | sure | near |
| shark | acorn | boast | amuse |
| camel | snail | greed | haste |
| whale | grape | smash | shrug |
| tiger | thorn | quest | frail |
| truck | berry | agony | bland |
| flood | pearl | gloom | weird |
| piano | olive | eager | trick |
| ocean | lemon | glory | pause |
| storm | mouse | brave | trace |
| giant | shell | panic | tight |
| tower | crown | pride | minor |
| beach | phone | anger | brief |
| horse | drink | shock | least |
| train | smile | truth | quiet |
| river | radio | heavy | piece |
| hotel | paper | trust | short |
| walrus | dimple | marvel | prefix |
| rocket | pebble | thrill | rumour |
| cannon | walnut | brutal | scarce |
| circus | tomato | admire | humble |
| dragon | bullet | genius | polite |
| jungle | insect | misery | remark |
| parade | cherry | terror | bother |
| ladder | pencil | wisdom | excuse |
| cattle | needle | virtue | assist |
| valley | button | honour | phrase |
| palace | ticket | empire | aspect |
| castle | pocket | wealth | unique |
| museum | coffee | crisis | narrow |
| forest | letter | career | minute |
| typhoon | freckle | prosper | thrifty |
| giraffe | thimble | gallant | flutter |
| gorilla | sparrow | torment | pronoun |
| tornado | emerald | expanse | flicker |
| volcano | apricot | dynasty | literal |
| tractor | biscuit | eternal | impulse |
| mansion | vitamin | miracle | glimpse |
| rainbow | diamond | liberty | portion |
| harbour | battery | courage | episode |
| factory | matches | passion | shallow |
| bookcase | tweezers | demolish | cowardly |
| windmill | ladybird | infinity | mischief |
| dinosaur | bracelet | epidemic | syllable |
| monument | mosquito | devotion | reminder |
| elephant | ornament | paradise | intimate |
| mountain | chestnut | ambition | instance |
| audience | magazine | disaster | incident |
