# Supplementary material for: Semantic Size of Abstract Concepts: It Gets Emotional When You Can’t See It
Source: PLoS One. 2013 Sep 25;8(9):e75000. doi: 10.1371/journal.pone.0075000 (PMC3783453; doi:10.1371/journal.pone.0075000)
Supplement: Table S2 — The five rating scales. For each scale, the definition of each variable given in the instructions and the associated labels (from left to right) of each rating scale are indicated. (DOCX) [file pone.0075000.s002.docx]

**Table S2. The five rating scales. For each scale, the definition of each variable given in the instructions and the associated labels (from left to right) of each rating scale are indicated.**

| **Concreteness** | |  |
| --- | --- | --- |
|  | **Description** | Concreteness is a measure of how concrete or abstract something is. A word is CONCRETE if it represents something that exists in a definite physical form in the real world. In contrast, a word is ABSTRACT if it represents more of a concept or idea. |
|  | **Labels** | Very abstract - Neither especially abstract nor concrete - Very concrete |
|  |  |  |
| **Semantic Size** | |  |
|  | **Description** | Size is a measure of something’s dimensions, magnitude, or extent. A word represents something BIG if it refers to things or concepts that are large. A word represents something SMALL if it refers to things or concepts that are little. |
|  | **Labels** | Very small - Neither small nor big - Very big |
|  |  |  |
| **Emotional Arousal** | |  |
|  | **Description** | Arousal is a measure of excitement versus calmness. A word is AROUSING if it makes you feel stimulated, excited, frenzied, jittery, or wide awake. A word is UNAROUSING if it makes you feel relaxed, calm, sluggish, dull, or sleepy. |
|  | **Labels** | Very unarousing - Moderately arousing - Very arousing |
|  |  |  |
| **Emotional Valence** | |  |
|  | **Description** | Valence is a measure of value or worth. A word is POSITIVE if it represents something considered good, whereas a word is NEGATIVE if it represents something considered bad. |
|  | **Labels** | Very negative - Neutral - Very positive |
|  |  |  |
| **Age of Acquisition** | |  |
|  | **Description** | A word’s age of acquisition is the age at which that word was initially learned. Please estimate when in your life you think you first acquired or learned each word. That is, try to remember how old you were when you learned each word either in its spoken or written form (whichever came first). |
|  | **Labels** | 0 - 2 - 4 - 6 - 8 - 10 - 12 - 13+ |
