# Supplementary material for: Semantic Size of Abstract Concepts: It Gets Emotional When You Can’t See It
Source: PLoS One. 2013 Sep 25;8(9):e75000. doi: 10.1371/journal.pone.0075000 (PMC3783453; doi:10.1371/journal.pone.0075000)
Supplement: Procedure S1 — Word rating task. (DOCX) [file pone.0075000.s003.docx]

**Procedure S1. Word rating task.**

An independent sample of 16 native English speakers was recruited to provide ratings on the following 5 variables related to our stimulus words: Concreteness, Semantic Size, Emotional Arousal, Emotional Valence, and Age of Acquisition (AoA). Each scale took approximately 10 min to complete and participants received £6 compensation.

Participants were seated in front of a PC. They were given a description of each scale before providing ratings for that variable and these are presented in **Table S2**. For each scale, the entire list of words was presented in a random order. A visual analogue scale (VAS) was employed for collecting responses. Participants were instructed to use the mouse to slide a vertically-oriented arrow across a horizontal line scale and indicate the position that best fit their rating by clicking the mouse. The two ends as well as the midpoint of each scale were labelled in the space below the line. For AoA, additional labels were used (see **Table S2**). On each trial, the arrow’s initial position was set to the midpoint of the line.

Resulting ratings were coded as integer values from 0 to 100. These are summarized by condition in **Table 1**. Emotional Valence is reported in two ways. Raw Valence is the average of the original ratings which ranged from 0 (very emotionally negative) to 100 (very emotionally positive). The overall Raw Valence, however, does not convey the degree of emotionality (i.e., a combination of highly positively and negatively rated words can result in the same mean as that of somewhat positively and negatively rated words). Absolute Valence was used to represent the extent of unsigned valence and, as such, resembles Emotional Arousal (see, e.g., Bradley & Lang, 1999). To calculate Absolute Valence, Emotional Valence ratings were re-centered around 0 (a 100-point scale from -50 to +50) and their absolute value taken and doubled in order to re-scale the values onto a 100-point range.
